# Case report: Successful therapy with recombinant human vascular endostatin in an elderly man with colon telangiectasia and idiopathic thrombocytopenic purpura

**DOI:** 10.3389/fmed.2024.1511513

**Published:** 2025-01-15

**Authors:** Yushan Wu, Yongbin Jia, Mingshan Jiang, Hu Zhang

**Affiliations:** ^1^Department of Gastroenterology, West China Hospital, Sichuan University, Chengdu, China; ^2^Centre for Inflammatory Bowel Disease, West China Hospital, Sichuan University, Chengdu, China; ^3^Lab of Inflammatory Bowel Disease, Frontiers Science Center for Disease-Related Molecular Network, West China Hospital, Sichuan University, Chengdu, China

**Keywords:** gastrointestinal bleeding, telangiectasia, colon, idiopathic thrombocytopenic purpura, recombinant human vascular endostatin, vascular endothelial growth factor

## Abstract

An 83-year-old male presented to our Digestive System Department with a 5-day history of severe gastrointestinal (GI) bleeding and a 14-year history of idiopathic thrombocytopenic purpura (ITP) with low platelet levels. Colonoscopy revealed extensive telangiectasias throughout the colon, particularly in the transverse and ascending segments. Standard treatment with proton-pump inhibitors and somatostatin proved ineffective. Additionally, conventional therapies such as estrogen and thalidomide were contraindicated due to the comorbidity of ITP. Endoscopic hemostasis was also difficult to perform because of the widespread nature of the lesions. However, after the innovative use of four courses of recombinant human vascular endostatin (Endostar) therapy, the colon telangiectasia was completely resolved, and the patient reported no GI bleeding for 2 years. Managing severe GI bleeding with a rare etiology is particularly challenging, especially in patients with contraindications to conventional treatments due to comorbidities. In this case, a vascular endothelial growth factor (VEGF) inhibitor was successfully applied to treat a refractory and rare GI bleeding, which may offer a novel therapeutic approach for similar cases.

## 1 Introduction

Colon telangiectasia refers to a group of colonic vascular malformations. The terms vascular malformation (VM) and angiodysplasia (AD) are often used interchangeably with colon telangiectasia. It can occur as an independent disease or as a manifestation of systemic conditions involving the gastrointestinal tract. Gastrointestinal vascular abnormalities are one of causes of unexplained gastrointestinal bleeding. Angiogenesis, the formation of new capillaries from preexisting blood vessels, plays a critical role in both physiological and pathological conditions. This process is regulated by multifunctional cytokines such as transforming growth factor-β1 (TGF-β1) and vascular endothelial growth factor (VEGF) (1). Pathologically, colon telangiectasia is closely associated with increased VEGF expression, which promotes endothelial cell proliferation and migration. This overexpression may result in abnormal blood vessel formation, increasing the risk of gastrointestinal bleeding (2).

The etiology of colon telangiectasia includes both hereditary and acquired factors, such as hereditary hemorrhagic telangiectasia (HHT) (3). The disease course is often prolonged, with lesions typically detected during endoscopic evaluation prompted by anemia or symptoms of gastrointestinal bleeding, such as hematemesis or melena. Currently, no standardized treatment guidelines exist for colon telangiectasia. Management options include drug therapy, endoscopic intervention, interventional radiology, and surgical treatment. Among these, conventional hemostatic drugs are commonly employed but often show limited efficacy. With advances in digestive endoscopy, endoscopic treatment has become the mainstay for managing bleeding lesions. For diffuse vascular abnormalities, particularly angiodysplasia, alternative drug therapies such as estrogen/progestin combinations (4), thalidomide, and somatostatin may be considered when endoscopic or surgical options prove ineffective. Thalidomide and somatostatin have demonstrated efficacy through antiangiogenic mechanisms in previous studies (5, 6). In moderate to severe HHT-associated gastrointestinal bleeding, bevacizumab and other antiangiogenic drugs have been recommended (7). Small, uncontrolled studies have also shown systemic antiangiogenic therapies can alleviate anemia, reduce transfusion requirements, and improve quality of life (7–9).

Antiangiogenic drugs are approved globally for various tumors. These agents primarily inhibit neovascularization by targeting endothelial cell proliferation and migration. Antiangiogenic therapies encompass macromolecular monoclonal antibodies targeting VEGF/VEGF receptors, multi-targeted small-molecule tyrosine kinase inhibitors (TKIs), and broad-spectrum recombinant human endostatin. Recombinant human endostatin (Endostar) is an intravenous antiangiogenic drug widely used off-label for conditions beyond non-small cell lung cancer, including melanoma, osteosarcoma, nasopharyngeal carcinoma, breast cancer, and esophageal cancer. It inhibits angiogenesis through multiple pathways, such as reducing VEGFR-2 expression and preventing VEGF-induced receptor phosphorylation (10, 11). Studies indicate its ability to inhibit angiogenesis and lymphangiogenesis in colorectal cancer by suppressing VEGF-A, VEGF-C, and VEGF-D expression (12–14).

Based on the complexity of this case and current guidelines for similar diseases, recombinant human endostatin was considered. This case highlights the interplay between colon telangiectasia and idiopathic thrombocytopenic purpura (ITP), emphasizing innovative therapeutic approaches for complex cases.

## 2 Case presentation

An 83-year-old male patient was admitted to our Digestive System Department with a 5-day history of profuse melena, accompanied by dizziness and fatigue. The patient denied fever, abdominal pain, or heartburn. He had been diagnosed with idiopathic thrombocytopenic purpura (ITP) at the age of 69 and had been intermittently treated with corticosteroids, which increased his risk of gastrointestinal (GI) bleeding and further complicated the clinical scenario. After thorough medical history questioning, the patient had no prior history of gastrointestinal bleeding, did not require blood transfusion, and had not undergone gastroscopy or colonoscopy before this admission.

**Figure 1:**
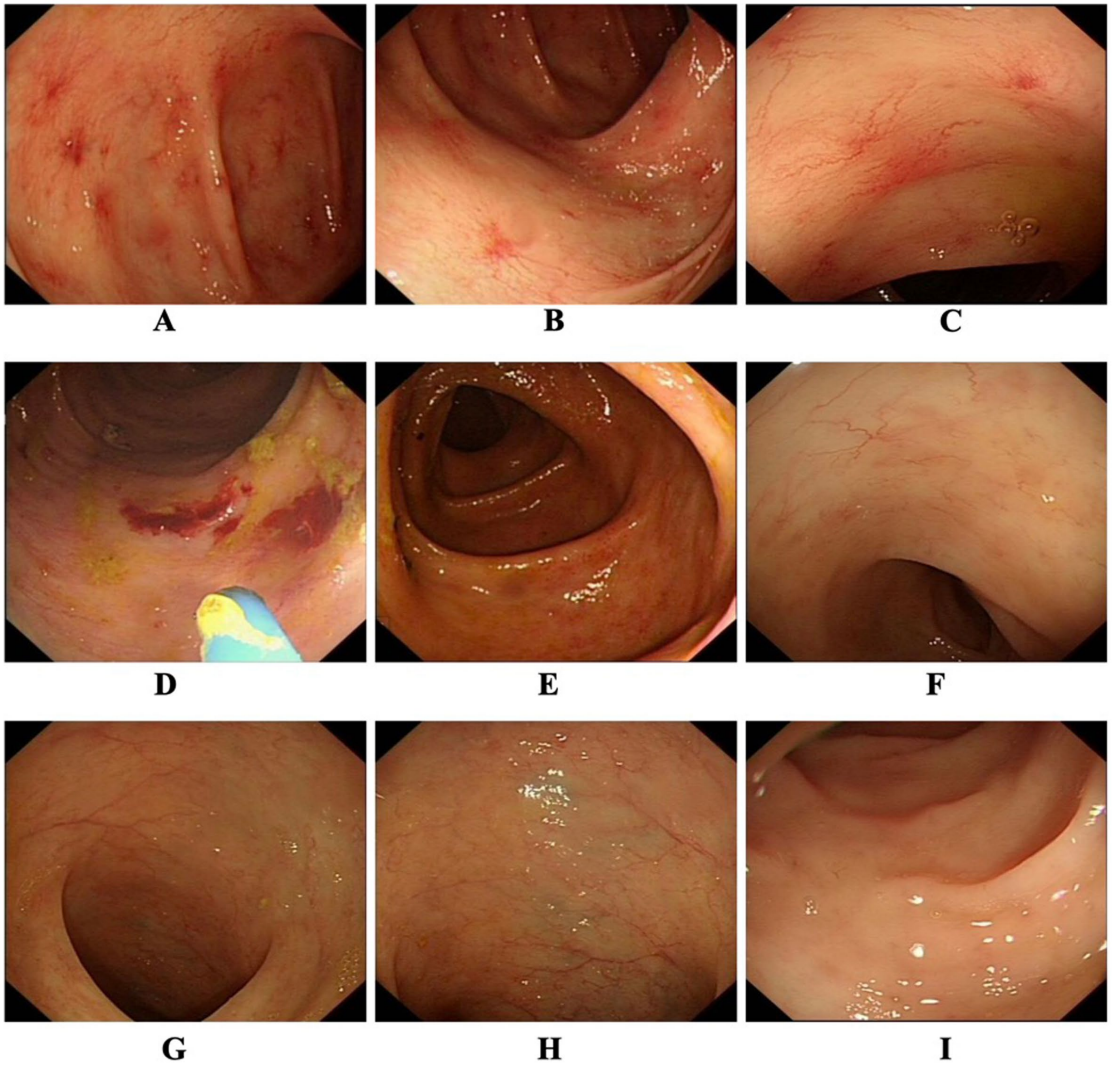
**(A–C)** First colonoscopy images before treatment showed that multiple telangiectasias were found, especially in the transverse colon and the ascending colon. **(D–F)** After two courses of Endostar (20 days later), multiple telangiectasias were still observed in the transverse colon and the ascending colon during the colonoscopy, but the magnitude and extent had decreased. **(G–I)** After four courses of Endostar therapy, the colonoscopy showed that no telangiectasias were found in any segment of the colon.

Upon admission, laboratory tests indicated iron-deficiency anemia, with a hemoglobin level of 6.1 g/dL and a platelet count of 25 × 10^9^/L. A colonoscopy was performed to determine the source of the hemorrhage, revealing multiple telangiectasias, primarily located in the transverse and ascending colon (Figures 1A–C). After blood transfusion therapy, the patient’s hemoglobin level increased to 7 g/dL. Despite continuous treatment with proton-pump inhibitors and somatostatin, the patient continued to report episodes of melena and a persistently low platelet count during hospitalization.

Performing endoscopic hemostasis posed significant challenges due to the widespread and diffuse nature of the lesions. Alternative therapies, including estrogen, thalidomide, and recombinant human vascular endostatin (Endostar), were considered. Given the patient’s advanced age, low platelet count, and the potential risks associated with thrombopoiesis and other adverse effects, estrogen and thalidomide were ruled out. After extensive multidisciplinary discussions and approval by the hospital’s ethics committee, we opted for the “off-label” use of Endostar. The drug was administered at a dosage of 0.25 mg/kg/day, with each course lasting 10 days (150 mg per course), repeated monthly. In order to ensure patient safety, we concurrently administered tranexamic acid and carbazochrome sodium sulfonate for hemostasis during the first course of Endostar treatment, alongside parenteral nutrition support. During four courses of Endostar treatment, we progressively adjusted the patient’s diet and used proton pump inhibitor, medications to repair intestinal mucosa, and drugs to promote platelet production. Additionally, iron supplements, recombinant human erythropoietin, and probiotics were provided to support recovery. Electrolyte homeostasis was maintained. And regular monitoring of routine blood tests, liver and kidney function, electrolytes, coagulation parameters, and fecal occult blood tests was conducted to detect any worsening of gastrointestinal bleeding or the emergence of other complications and adverse reactions.

**Figure 2:**
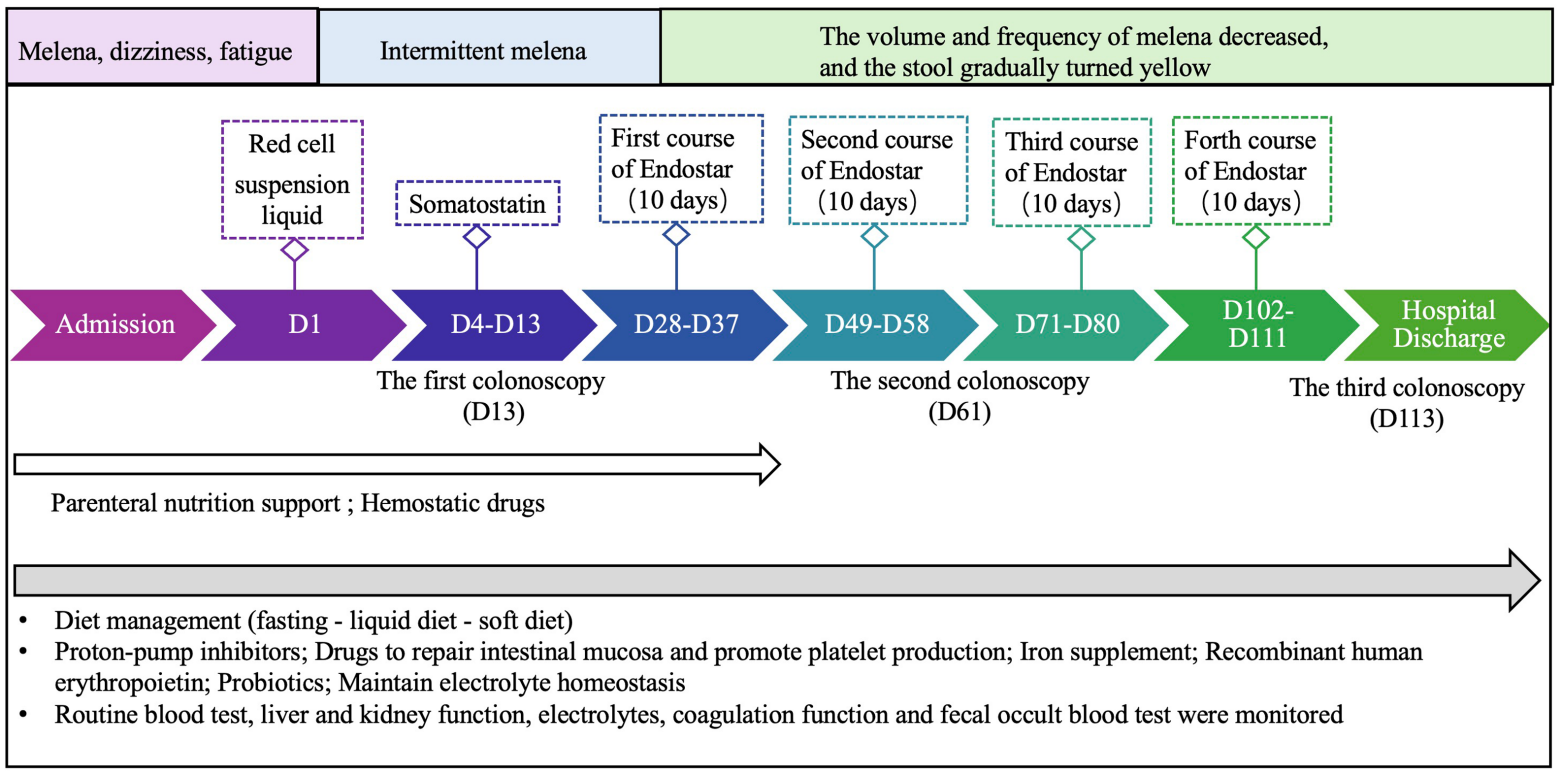
A timeline of test and treatment.

**Figure 3:**
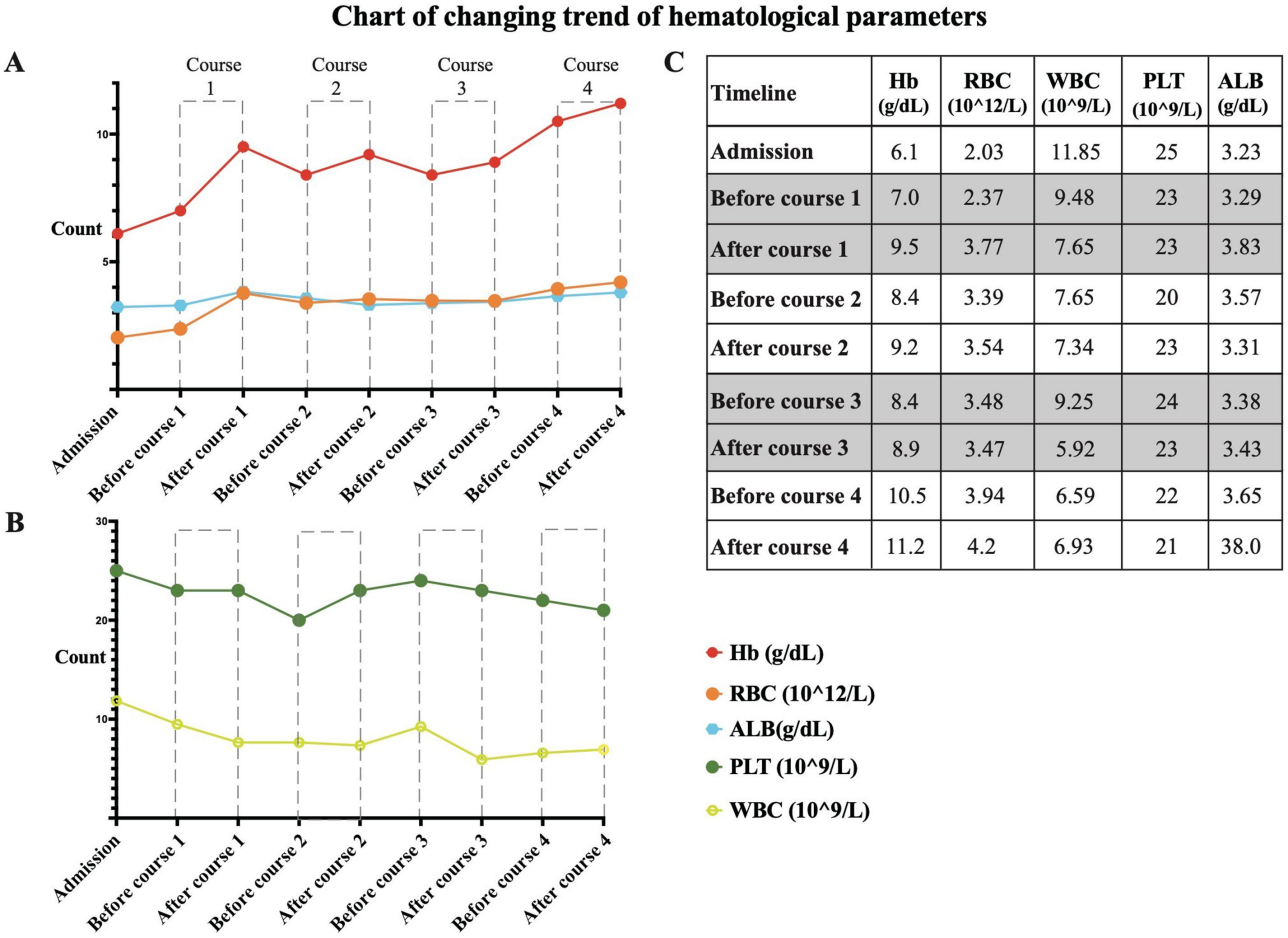
Key hematologic parameters from admission to discharge. **(A)** The trends in hemoglobin (Hb) concentration, red blood cell (RBC) count, and albumin (ALB) concentration. **(B)** The trends in platelet (PLT) count and white blood cell (WBC) count. **(C)** A graph of specific values corresponding to the figure.

A review of the diagnosis and treatment timeline is presented in Figure 2. On admission, the vital signs of the patient were relatively stable, with a blood pressure of 138/65 mmHg, a temperature of 36.5 ° C, a heart rate of 89 beats per minute, and a respiratory rate of 19 breaths per minute. During treatment, the lowest blood pressure was 104/65 mmHg. After the last Endostar infusion, the patient’s blood pressure was 122/60 mmHg before discharge. Of note, the patient had a previous history of grade 2 hypertension, so although his blood pressure was within the normal range, it was alarming. Key hematologic variables from admission to discharge are shown in Figure 3. After each course of Endostar treatment, the hemoglobin and red blood cell count of patients had increased to varying degrees, and the overall trend was on the rise, which indicates the effectiveness of Endostar to a certain extent. At the same time, there were no obvious abnormalities in liver function, renal function, coagulation function and inflammatory indicators during treatment, indicating the safety of Endostar.

After two courses of Endostar, a follow-up colonoscopy was performed to assess the treatment’s effectiveness. The results showed that while colonic telangiectasias were still present, the magnitude and extent had decreased (Figures 1D–F). Additionally, an erosive bleeding lesion without colonic dilatation was identified and successfully treated with argon plasma coagulation (APC) (Figure 1D). After completing four courses of Endostar over a 4-month hospitalization, the patient’s gastrointestinal symptoms improved significantly. A subsequent colonoscopy showed complete resolution of the telangiectasias in all segments of the colon (Figures 1G–I). At the time of discharge, the patient’s hemoglobin level had risen to 11.2 g/dL. During follow-up visits, the patient reported no recurrence of melena for 2 years.

## 3 Discussion

The patient presented with massive melena as the primary symptom, gastrointestinal bleeding may originate from the upper gastrointestinal tract or small intestine. A gastroscopy was performed, which showed no bleeding sites or telangiectasias. Given the patient’s advanced age, low platelet count, and poor tolerance, double-balloon enteroscopy was not performed. Additionally, the patient declined capsule endoscopy due to its high cost and the potential risk of retention. It is significant for the diagnosis and treatment to clarify the extent, degree and bleeding of the lesions in the upper gastrointestinal tract and small intestine. If a similar case occurs, gastroscopy and double-balloon enteroscopy, or intestinal imaging should be performed if the patient’s condition permits, after comprehensive judgment based on the patient’s age, cardiopulmonary function, comorbidities, and economic status.

**Figure 4:**
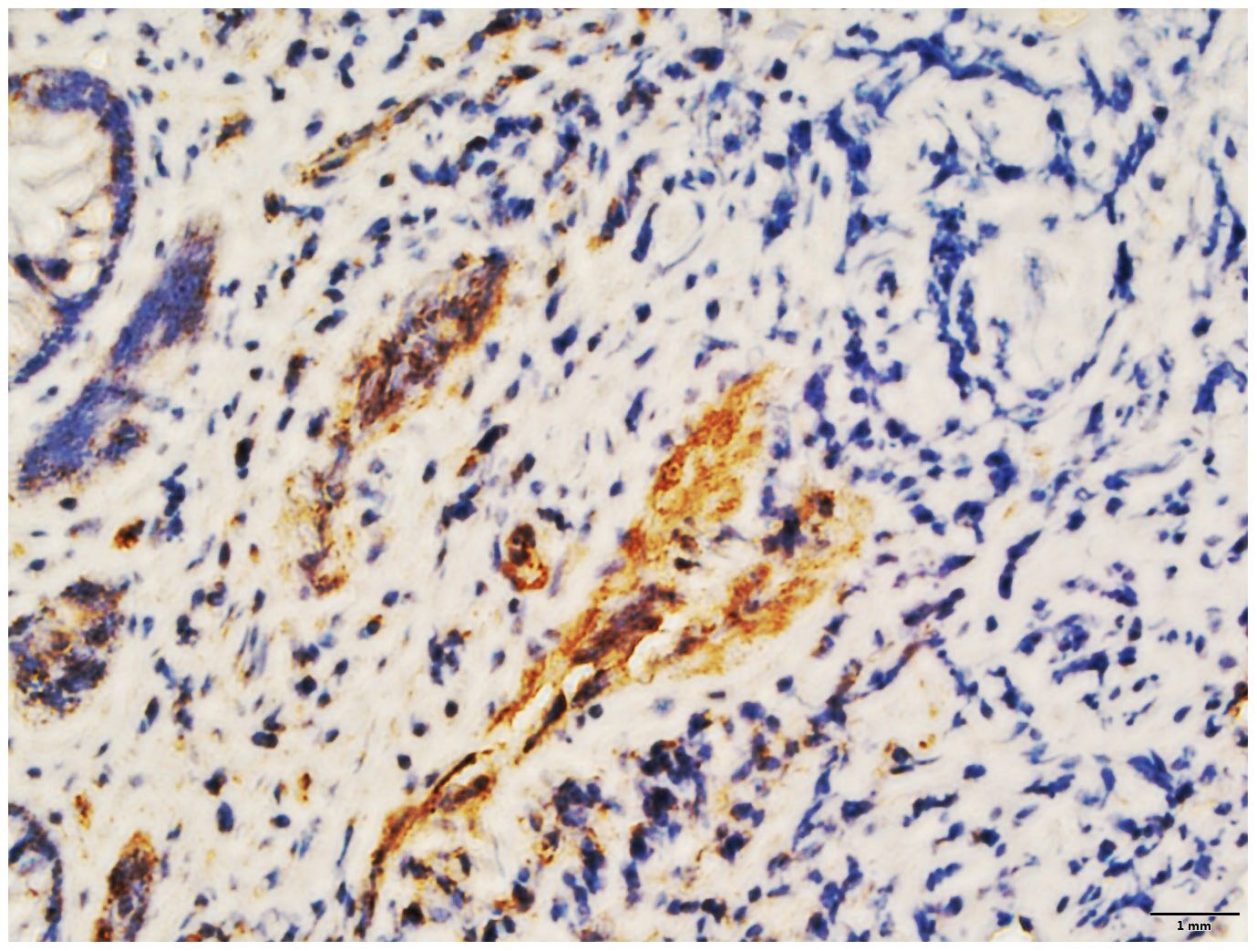
The colon tissue of the patient exhibited elevated levels of vascular endothelial growth factor (VEGF) as determined by immunohistochemical analysis.

Despite transfusions of red blood cell suspension and administration of somatostatin and hemostatic drugs, recurrent melena persisted. Given the patient’s chronic thrombocytopenia and increased thrombosis risk during prolonged hospitalization, recombinant human endostatin was selected over estrogen/progestin therapy or thalidomide to minimize complications such as bone marrow suppression or thrombosis. Immunohistochemical analysis confirmed high VEGF expression in colonic tissues, supporting the use of antiangiogenic therapy (Figure 4).

Following two courses of Endostar, colon telangiectasia persisted but resolved after four courses. Pharmacokinetic studies in oncology indicate significant interindividual variability in drug concentration curves, with trough concentrations increasing with successive infusions (15). Monitoring drug concentrations could optimize dosing and improve outcomes in future cases.

Bevacizumab, another antiangiogenic agent, functions as a recombinant humanized monoclonal antibody targeting VEGF. Bevacizumab is effective in various malignancies and gastrointestinal bleeding associated with HHT (8, 16). In addition, long-term bevacizumab was used to successfully treat a patient with glanzmann’s thrombasthenia and recurrent digestive bleeding due to gastrointestinal angiodysplastic lesions (17). However, it carries higher risks of bleeding, hypertension, and delayed wound healing compared to Endostar. Recombinant human endostatin, on the other hand, exhibits a favorable safety profile, with cardiac toxicity (15), occasional diarrhea and abnormal liver function (18). Among them, the gastrointestinal adverse reactions are mostly mild and moderate, which can be tolerated by most patients and can be relieved after appropriate treatment. It has been reported that recombinant human endostatin can reduce blood pressure through NO production (19). But it is worth noting there is a risk of hypotension with massive GI bleeding. Close monitoring of blood pressure, urine output, and mental state mitigated potential risks during treatment. From another perspective, whether the mechanism of blood pressure lowering by endostatin played a role in hemostasis in this case warrants further exploration.

This case underscores the challenges in managing digestive vascular malformations and highlights antiangiogenic therapy as a viable option for severe, refractory bleeding. The advanced age, extensive lesions, and comorbidities added complexity to treatment, but Endostar provided significant efficacy, avoiding surgery and associated complications.

For similar cases, careful consideration of bleeding etiology, clinical history, and drug reactions is essential. Conventional treatments must be evaluated for efficacy, and the root cause of bleeding must be meticulously identified to prevent severe complications from inappropriate therapies.

## 4 Conclusion

VEGF plays a critical role in angiogenesis. The innovative use of recombinant human vascular endostatin (Endostar), a broad-spectrum anti-angiogenic agent, demonstrates a promising therapeutic strategy in managing colon telangiectasia and may provide a new treatment avenue for similar cases where standard interventions fail.

## Data Availability

The original contributions presented in the study are included in the article/Supplementary material, further inquiries can be directed to the corresponding author.
